# Revisiting the variation of clustering coefficient of biological networks suggests new modular structure

**DOI:** 10.1186/1752-0509-6-34

**Published:** 2012-05-01

**Authors:** Dapeng Hao, Cong Ren, Chuanxing Li

**Affiliations:** 1 College of Bioinformatics Science and Technology, Harbin Medical University, Harbin, 150081, China; 2 The Second Department of Orthopedics, the Second Affiliated Hospital of Harbin Medical University, Harbin, 150081, China

## Abstract

**Background:**

A central idea in biology is the hierarchical organization of cellular processes. A commonly used method to identify the hierarchical modular organization of network relies on detecting a global signature known as variation of clustering coefficient (so-called modularity scaling). Although several studies have suggested other possible origins of this signature, it is still widely used nowadays to identify hierarchical modularity, especially in the analysis of biological networks. Therefore, a further and systematical investigation of this signature for different types of biological networks is necessary.

**Results:**

We analyzed a variety of biological networks and found that the commonly used signature of hierarchical modularity is actually the reflection of spoke-like topology, suggesting a different view of network architecture. We proved that the existence of super-hubs is the origin that the clustering coefficient of a node follows a particular scaling law with degree k in metabolic networks. To study the modularity of biological networks, we systematically investigated the relationship between repulsion of hubs and variation of clustering coefficient. We provided direct evidences for repulsion between hubs being the underlying origin of the variation of clustering coefficient, and found that for biological networks having no anti-correlation between hubs, such as gene co-expression network, the clustering coefficient doesn’t show dependence of degree.

**Conclusions:**

Here we have shown that the variation of clustering coefficient is neither sufficient nor exclusive for a network to be hierarchical. Our results suggest the existence of spoke-like modules as opposed to “deterministic model” of hierarchical modularity, and suggest the need to reconsider the organizational principle of biological hierarchy.

## Background

The high relevance between functional organization and topological features has motivated the development of statistical measures to characterize cellular networks. Increasingly, these measures reveal that biological network organization is characterized by the power law of degree distribution, the concept of modularity and the degree correlations on connected nodes [1-3]. Networks with high modularity have dense connections between the nodes within same cellular functions but sparse connections between nodes in different functions. Furthermore, a central theory in biology is the hierarchical organization of cellular processes, which means that high-level processes are build by connecting low-level ones [4,5]. For example, the process mitosis is composed by several low-level functions, such as spindle assembly, centrosome separation and chromosome alignment. Consequently, it is reasonable to suppose that functional modules of interest are hierarchically organized in the same way, that small modules are combined into larger modules and then further combined into even larger ones. This complexity, therefore, poses great challenges to researchers trying to understand the modularity structure of cellular networks.

To identify the hierarchical modularity of metabolic networks, Ravasz et al. focused on detecting a “global signature” of network architecture [6,7]. In Ravasz’s study, they revealed that for metabolic networks and for certain hierarchical networks the clustering coefficient, *C*(*k*), of a node follows a scaling law with degree *k C*(*k*) ~ *k*^-1^. To explain this, they proposed a network model which possesses both the power law of degree distribution and the scaling law of *C*(*k*). The starting point of this network model is a small cluster of five fully connected nodes; then creates four identical replicas, connecting the peripheral nodes of each cluster to the central node of the old cluster, resulted in a large 25-node cluster. Next, four replicas of this 25-node cluster are generated and the 16 peripheral nodes are connected to the central node of the old cluster, obtaining a larger cluster of 125 nodes. These replication and connection steps can be repeated indefinitely to generate a hierarchical architecture. In each step *i*, the number of nodes in the network is 5^*i*^. This network model, which we explicitly denote by “deterministic hierarchical model”, has subsequently a great influence on the studies of network biology [8,9], and the scaling of *C*(*k*) is widely used to identify whether or not a network is hierarchically organized nowadays.

Two former studies have suggested that the decrease of *C*(*k*) can be tentatively attributed to the tendency that large degree nodes are connected to small degree ones in biological networks[1,10]. For example, Soffer and Vazquez proposed a novel measurement of clustering coefficient taking into account of the neighborhood degree of node, which didn’t scale with *k*. Their work suggested that the variation of *C*(*k*) can be attributed to neighborhood degree distribution. However, the “deterministic model” is also anti-correlated. Thus, it is still possible that both the degree anti-correlation and the variation of *C*(*k*) is the reflection of hierarchy, suggesting that proper “null model” is needed to clarify their relationships. Moreover, metabolic networks is nicely approximated by *C*(*k*) ~ *k*^-1^, providing a strong evidence for the existence of hierarchy in these networks. However, to our best knowledge, former studies didn’t directly indicated why *C*(*k*) strictly follows this scaling law (*k*^-1^) in metabolic networks. These may be the reasons why the variation of *C*(*k*) is still widely used in assessing biological network hierarchy. In fact, almost every study on biological networks that observed the variation of *C*(*k*), including protein-protein networks, functional networks, human disease networks or even ecological networks, claimed that they have found a hierarchical modular structure, for example [11-17]. This situation suggested that, a further and systematical investigation of clustering coefficient focused on different types of biological networks is necessary. In this work we revealed the reason why *C*(*k*) scales with *k*^-1^ in metabolic networks and suggested by “null model” that the variation of *C*(*k*) is neither sufficient nor exclusive for a hierarchical network. Our findings suggest the existence of spoke-like topology as opposed to “deterministic hierarchical model”.

## Results and Discussion

### Origin of the scaling law in metabolic networks

**Figure 1:**
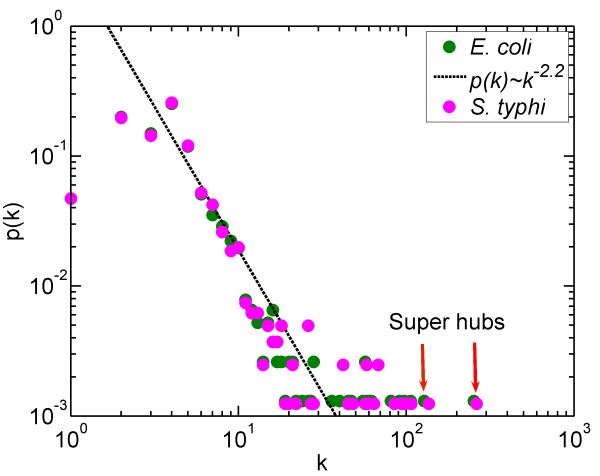
**The degree distribution of two metabolic networks.** For each network (*E.coli* and *S.typhi*, colored by green and pink dot respectively), the two largest super-hubs are pointed out.

**Figure 2:**
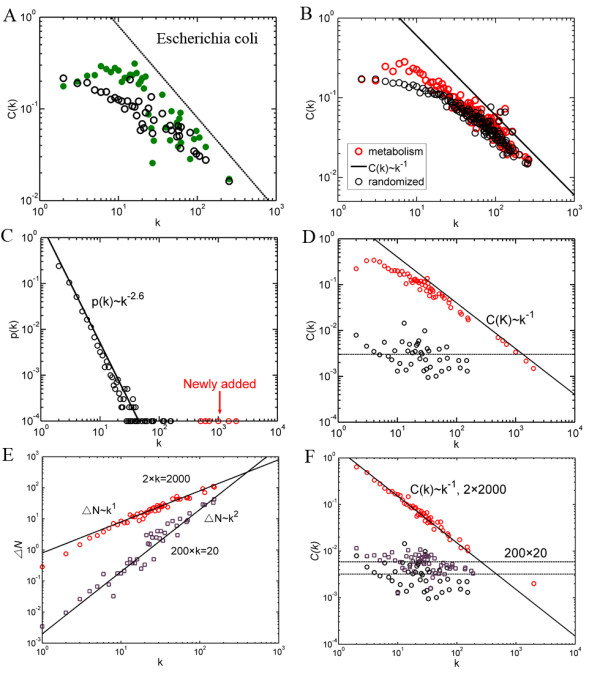
**Origin of the scaling law of*C*(*k*).** (**A**) *C*(*k*) for metabolic network of *E.coli* (green dot) and a randomized network (black circle). (**B**) The *C*(*k*) curves averaged over 43 organisms (red circle) and over 43 randomized networks (black circle). (**C**) The degree distribution of a random network with 4 super-hubs added. (**D**) The variation of *C*(*k*) of the random network before and after 4 super-hubs added (corresponds to black and red circles respectively). (**E**) Number of newly added triangles, *△N* as a function of *k*. (**F**) *C*(*k*) for random networks with large degree nodes added. In (E) and (F), black circles correspond to the original network, red circles correspond to the network after 2 nodes with degree 2,000 added and purple squares corresponds to the network after 200 nodes with degree 20 added.

We start by indicating why clustering coefficient distribution of metabolic networks strictly follows the particular scaling law (*k*^-1^). The clustering coefficient, defined as C(k) = 2N/k(k-1) , provides a measure of the level of interconnectivity in the neighborhood of a node, where N is number of triangles formed by the node and a link between any two direct neighbors of it [7]. In the former study, Ravasz et al. found a scaling law of *C*(*k*). They argued that this scaling law was not expected for a random scale-free network of similar sizes, indicating the absence of hierarchy in random networks. In the study, they used the B-A model to generate random scale-free networks [2]. One problem with their random network model, however, is that it does not take into account the existence of so-called super-hubs in metabolic networks (i.e. ATP and H_2_O). Drawing the degree distribution of metabolic networks shows the existence of “super hubs” that are unexpected from the approximated power-law degree distribution (Figure 1). A single super hub can have great impact on topological measures of network density such as clustering coefficient, as it carries a lot of edges. To take into account the super-hubs, here we generate random networks by randomly rewiring the links of metabolic networks, which preserves the same degree distribution [1]. We plot the *C*(*k*) curve of *E.coli* and the curves averaged over the metabolic networks of all 43 organisms, along with their randomized counterparts (Figure 2A and 2B). As can be seen, inconsistent with the former study, the metabolic networks and random networks show similar dependence of clustering coefficient on node’s degree. The only property shared by metabolic networks and their random counterparts is the degree sequence, while the prominent feature of the degree sequence of metabolic networks is the existence of super-hubs. Therefore, this result suggests that super hubs is probably the reason why *C*(*k*) scales with *k*^-1^. Note that the metabolic networks and random networks are separated when k is low, suggesting that metabolic networks have relatively higher modular level than random networks. However, this difference cannot be used as an evidence of hierarchy.

One may argue that given the degree distribution, hierarchical structure of a network is largely defined, so it is not surprising that random networks generated this way have similar dependence of clustering coefficient on node’s degree. To rule out this possibility, we next investigate whether the scaling law of *C*(*k*) can be reproduced in a totally random network. For this purpose, we first generated a random scale-free network of 10,000 nodes with degree following *P*(*k*) ~ *k*^-2.6^, and then added several large degree nodes unexpected from the degree distribution (Figure 2C, using preferentially attachment). As shown in Figure 2D, *C*(*k*) of the network with several large degree nodes added appears to scale with *k*^-1^, as opposed to the original network that has no variation. Therefore, several super-hubs is sufficient to give rise to the scaling law of *C*(*k*). This result is reasonable. For example, the metabolic network of *E.coli* has 2,409 edges with average claustering coefficient $\bar{C}=0.21$ and degree of the largest hub k=253. To keep the same value of clustering coefficient for this super hub, its neighbors have to be connected by $n=k(k-1)*\bar{C}/2=6694$ edges, which is nearly 3 times of the number of edges in the network!

We then would like to present an analytical investigation for this result. Consider an undirected random network with *S* nodes, *M* edges and average clustering coefficient $\bar{C}$, the probability that a newly added node *j* has a link with a node *i* is $p_{\mathrm{ij}}=k_{i}/\sum_{s=1}^{S}k_{s}=k_{i}/2M$, and thus the expected number of edges that newly added node *j* connects to *i* is $m_{\mathrm{ij}}=\min\left(k_{j}.k_{i}/2M,1\right)$, where *k*_*i*_, *k*_*j*_ are the degrees for nodes *i* and *j* and the function min() is to make sure at most one edge connecting two nodes. In a random network with no degree correlation, the average degree of the neighbors of a node would be the average degree of the network, <*k*>. Thus, the expected number of edges that the newly added node connecting to a neighbor of node *i* is$m_{j<k>}=\min\left(k_{j}\cdot <k>/2M,1\right)$. The number of newly added triangles involving node *i* that generated by node *j* connecting to both node *i* and its neighbors can be roughly estimated by $\Delta N_{i}=k_{i}\cdot m_{\mathrm{ij}}\cdot m_{j<k>}$. For node *j* with small degree *k*_*j*_, $m_{\mathrm{ij}}$ takes the value $m_{\mathrm{ij}}=k_{j}\cdot k_{i}/2M$, and thus $\Delta N_{i}=m_{j<k>}\cdot \frac{k_{i}^{2}k_{j}}{2M}=\alpha_{j}k_{i}^{2}$, where $\alpha_{j}$ is determined by *k*_*j*_. Now the clustering coefficient of node *i* is $C'(k_{i})\approx \bar{C}+2\Delta N_{i}/k_{i}(k_{i}+1)\approx \bar{C}+2\alpha_{j}$, which doesn’t vary with degree *k*_*i*_. However, for a node with large *k*_*j*_, $m_{\mathrm{ij}}$ takes the value $m_{\mathrm{ij}}=1$ and thus $\Delta N_{i}=\alpha_{j}k_{i}^{}$. Considering that the clustering coefficient of a random scale-free network is extremely small ($\bar{C}\approx 0$, for example, there are thousands of triangles in biological networks, whereas there are only tens of triangles in random networks of similar size), the $C'(k_{i})$ is now mainly determined by $2\Delta N_{i}/k_{i}(k_{i}+1)$, thus $C'(k_{i})\text{\textasciitilde{}}k_{i}^{-1}$. To test this, we constructed a network with 10,000 nodes following the distribution *P*(*k*) ~ *k*^-2.6^, which has only 64 triangles in total and thus the $\bar{C}\approx 0$. Then we randomly added 2 nodes with degree 2,000 and 200 nodes with degree 20 into this network respectively, of which the number of newly added triangles ΔN as a function of degree *k* is counted (Figure 2E). Although the number of newly added edges is the same, the number of newly added triangles increases in different rates as a function of *k*: $\Delta N\sim k^{1}$in the first case and $\Delta N\sim k^{2}$ in the second case respectively! As a result, the clustering coefficient shows a perfect scaling dependence on node’s degree in the first case, whereas it doesn’t vary with *k* in the second case (Figure 2F). This striking difference comes from the restriction $m_{\mathrm{ij}}=\min\left(k_{j}\cdot k_{i}/2M,1\right)$. For nodes with small degrees, $m_{\mathrm{ij}}$ takes the value$m_{\mathrm{ij}}=k_{j}\cdot k_{i}/2M$, whereas for nodes with large degrees, $m_{\mathrm{ij}}$ takes the value $m_{\mathrm{ij}}=1$. Notably, this formula reflects the fact that there is at most one edge connecting two nodes in these biological networks. Hence, this formula implies that connections between large degree nodes in metabolic networks are highly suppressed, compared to a random network with no constraints on edge multiplicity. For example, the two largest hubs in metabolic network of *E.coli* would be connected by $m=k_{i}\cdot k_{j}/2M=253*128/(2\cdot 2409)=13.4$ edges without constraints on edge multiplicity! In this case, a large degree node is forced to connect to small degree ones; as a consequence, its clustering coefficient is relatively small.

It should be noted that the clustering coefficient in the first case is at least an order of magnitude larger than that of the network in the second case, suggesting that the existence of super-hubs is one of the origins of high clustering of metabolic networks. Thus, when the level of clustering coefficient is regarded as a measure of modularity level, the existence of super-hubs should be considered, otherwise the modularity level of biological networks would be highly overestimated [7,18].

### Variation of C(*k*) is a reflection of degree correlation

Next, we ask whether the existence of super-hubs is the only reason that biological networks show dependence of clustering coefficient on node’s degree. However, we found that for other biological networks, the *C*(*k*) curve can be highly different with random networks of same degree distribution (for example, the protein-protein interaction network and the genetic synthetic lethal network. Additional file 1: Figure S1), suggesting that the variation of *C*(*k*) cannot be solely attributed to the existence of super-hubs. For metabolic networks, we have shown that the dependence of clustering coefficient on node’s degree has its origin in the suppression of hub-hub connections ($m_{\mathrm{ij}}=\min\left(k_{j}\cdot k_{i}/2M,1\right)$). Hence, it is possible that even without the existence of super-hubs, the anti-correlation between hubs is enough to cause the variation of *C*(*k*). Former studies have found that many biological networks are disassortative, indicating that the strong repulsion between hubs is frequently observed [1,19-21].

**Figure 3:**
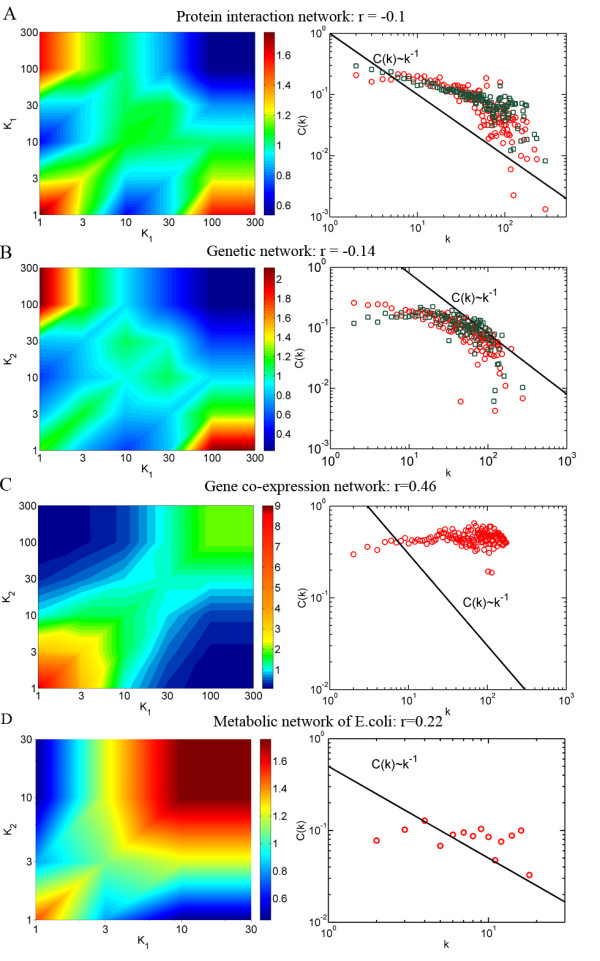
**Correlation profiles and the*C*(*k*) curves.** Correlation profile and *C*(*k*) curves of (**A**) protein network, (**B**) genetic network, (**C**) gene coexpression network and (**D**) metabolic network of *E.coli* with 21 currency metabolites (i.e. ATP and NADH) removed. Green rectangles in (A) and (B) are the *C*(*k*) curves of two simulated random networks.

To investigate the relationship between repulsion of hubs and the variation of *C*(*k*), we plotted the correlation profiles for biological networks, as well as their clustering coefficient distribution (Figure 3). The correlation profile compares the joint probability *P*(*k*_*i*_, *k*_*j*_) of finding a link between any two nodes of degree *k*_*i*_ and *k*_*j*_ with the same probability *P*_*r*_(*k*_*i*_, *k*_*j*_) in random counterparts [1,20]. The random counterparts are generated by randomly rewiring the links of original network, thus preserving the degree sequence. The correlation profiles for biological networks were generated by comparing with 100 randomized counterparts. The protein-protein interaction network and the genetic synthetic lethal network pose a higher level of anti-correlation: nodes with large degrees favor to link with nodes of small degrees (Figure 3A and 3B). A measure of degree correlation, known as assortative coefficient *r* (shown in the figure), is consistent with their correlation profiles [22]. Their corresponding clustering coefficient distributions show a decline with node’s degree, although with a clear deviation from scaling law *C*(*k*) ~ *k*^-1^. Note that there is a rapid decrease of *C*(*k*) for large node degree, corresponding to the highly suppressed region in the upper-right corner of correlation profiles (colored by dark blue). On the other hand, the gene co-expression network and the metabolic network with currency metabolites removed display a high level of hub affinity as opposed to anti-correlation; nodes with large degrees favor to link with other nodes of large degrees (Figure 3C and 3D). Their clustering coefficient distributions, therefore, do not decrease with node’s degree. Thus, the variation of *C*(*k*) perfectly coincides with the correlation profiles of network structure. This result is consistent with a former study of Soffer and Vazquez [10]. In their study, Soffer and Vazquez proposed a novel measurement of clustering coefficient of node that was normalized by its neighborhood degree, which didn’t show dependence on node’s degree. Their results also suggest that the degree correlation is probably the origin of the variation of *C*(*k*).

### Simulated annealing

**Figure 4:**
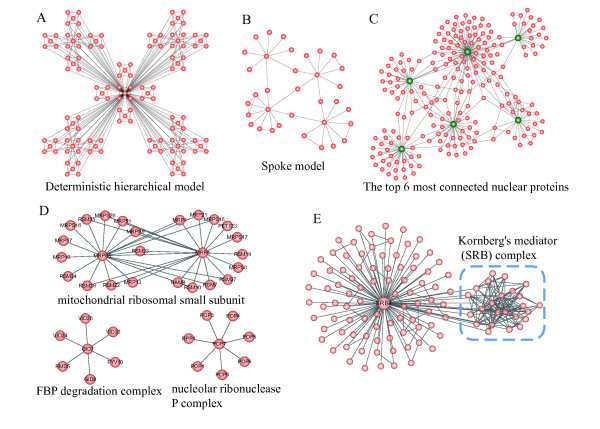
**Spoke modules.** (**A**) Deterministic hierarchical model. (**B**) Spoke model. (**C**) The connection of the top 6 best connected nuclear proteins and their neighbors displays a spoke-like topology. (**D**) Spoke-like protein complexes. (**E**) Gene *srb4* is a member of clique and a member of spoke-like topology.

However, it is still possible that both the anti-correlation between hubs and the variation of *C*(*k*) are reflections of hierarchy. A key procedure of generating “deterministic hierarchical model” is to connect peripheral nodes to the central node of a certain module and to avoid direct links between central nodes [7,23]. They provided little information about why this procedure is necessary. However, this procedure helps to give rise to both anti-correlation and the variation of *C*(*k*). For example, the assortative coefficient r=−0.077 for “deterministic hierarchical model” network of 5^6^ = 15625 nodes. To rule out the possible that both the anti-correlation and the variation of *C*(*k*) are reflections of hierarchy, one has to investigate whether a random network with similar level of repulsion between hubs would have the same variation of *C*(*k*). However, one problem with the random networks is that they are much less modular than biological networks. In other words, the neighbors of a same node are more likely to be linked in biological networks than in random networks (that is, more likely forming a triangle). To overcome this, we generated random networks by combining edge rewiring method and simulated annealing algorithm. We first generate seed networks that preserves the joint probability *P*(*k*_*i*_, *k*_*j*_) (see Materials and methods), and then conduct simulated annealing introducing an effective temperature *T* to globally minimize the following score function: $\text{E}=\left|{\text{N}}_{\text{random}}-\text{N}\right|/\text{N}$, where ${\text{N}}_{\text{random}}$is the number of triangles in random network and *N* is the number of triangles in real network. Random networks with the same level of modularity will have the minimum score 0. At each Monte Carlo step, we select two edges at random from this network and replace them with two new ones by rewiring them with a probability $\text{min }\left[\exp\left(-\Delta E/T\right),1\right]$, on one condition that the rewiring step preserves the joint probability *P*(*k*_*i*_, *k*_*j*_). Then, This Monte Carlo step is repeated until *E* achieves a stationary value. Because the minimum energy is given, it is easy to get a network with similar level of modularity as real network by inducing an appropriate temperature *T*. Figure 4 and B shows the *C*(*k*) curves for two random networks annealing to the two biological networks that show variation of *C*(*k*) (the green rectangles). As can be seen, the *C*(*k*) curves of the two random networks overlap with biological networks nicely. Since the seed networks are random networks, this result confirms that the dramatic decline of *C*(*k*) with node’s degree is the reflection of repulsion between hubs rather than reflection of rigid hierarchy as characterized by “deterministic hierarchical model”.

One concern is that a few of edges of the generated random networks and biological networks may be overlapped, and thus hierarchy structure is conserved in null networks. To rule out this possibility, we further generated much more stringent but uncorrelated random networks of which a large fraction of edges are overlapped with the biological network. However, we found that the variation of *C*(*k*) was substantially disappeared (Additional file 2: Figure S2). One should also note that the clustering coefficient distribution of Figure 3 shows clear deviation from any scaling law *C*(*k*) ~ *k*^-β^, further suggesting that biological networks cannot be characterized by the “deterministic hierarchical model”.

### Spoke versus “deterministic hierarchical model”

The “deterministic hierarchical model” suggests that the variation of *C*(*k*) is caused by rigid hierarchy that is built by connecting the external nodes of low-level dispersed modules to the central nodes of a high-level module.(Figure 4A) [23]. However, our results suggest that the variation of *C*(*k*) in biological networks, is caused by the abundance of large degree nodes connecting to those with much smaller degree, which we refer to as a heuristic “spoke” model (Figure 4B). The two models can be easily checked by visualizing the connection of a few hubs for a real network. Figure 4B shows the connection of the top 6 best connected proteins and their neighbors in a small protein interaction network formed by proteins localized in nucleus according to a high-confidence dataset (Figure 4C) [24]. Apparently, the protein network supports the picture of “spoke” model rather than rigid hierarchy of “deterministic hierarchical model”.

What do our results suggest for the conception of modularity? First of all, they suggest the existence of functional modules that are spoke-like or built by connecting spoke-like topologies. This new view will include many biological modules that can not be revealed by finding densely connected regions such as cliques or k-cores. For example, the functional module associated to cell wall organization is built by connecting several spokes (Additional file 3 Figure S3). Many biological pathways include enzymes and tens of its substrates may be better depicted by this view of modularity. We found that even protein complexes could be spoke-like as well. Figure 4D shows three protein complexes of *S. cerevisiae*, of which FBP degradation complex and nucleolar ribonuclease P complex are built by a single spoke, while mitochondrial ribosomal small subunit is built by connecting two spokes centered on *mrp4* and *mrps5* respectively. However, we stress that the traditional idea of modularity as finding densely connected regions is still useful in identifying cellular machines. In fact, the protein network integrates “spoke” topology and densely connected regions into a highly interconnected web. A single molecule could be both a member of clique and a member of spoke-like topology. For example, *srb4* encodes a core component of the SRB mediator complex of *S. cerevisiae* and is required for transcription of most yeast genes. However, the execution of the function of *srb4* also relies on the interaction of many poorly connected genes outside the complex such as *cbs1*, a mitochondrial translational activator of cob mRNA, resulting in a large spoke centered on *srb4* (Figure 4E). These explain why *C*(*k*) shows negative dependence on node’s degree in protein network, even though there are a large number of protein complexes.

### New hierarchical modularity paradigm

Finally, our work raises two fundamental questions: a question about motivation of spoke-like topology during evolution and a question about how low-level modules communicate with each other to generate high-level ones. A possible answer for the first question is that suppression between hubs confines mutational perturbations to the local. It is widely accepted that hub genes are more essential than poorly connected genes. Thus, the overabundance of spoke-like topology may reduce the accumulative effect of the mutational perturbations of two directly connected hubs. Another possible answer for the first question is that the overabundance of spoke-like topology shortens the distance between molecules, and thus signals propagate more quickly. A molecule connecting with a hub is more easily to propagate its signal than a molecule connecting with a poorly connected node. Given that most molecules of biological networks are poorly connected, this may be one of the reasons why these networks favor spoke-like topology. This speculation is supported by the finding that more nodes in an assortative network (i.e., social network) fail to connect to the largest component to propagate its signal than in a disassortative network (i.e., World Wide Web) [19,22].

**Figure 5:**
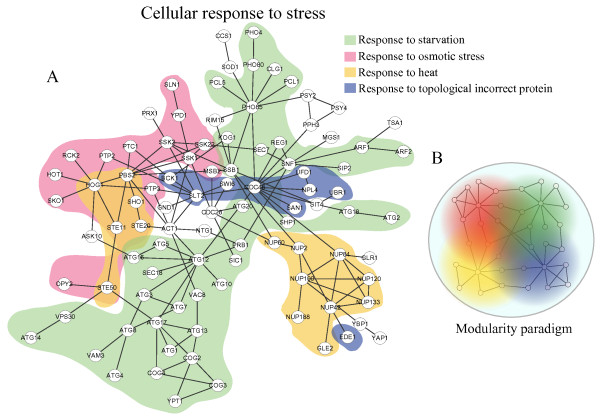
**New hierarchical modularity paradigm.** (**A**) The subnet related to cellular response to stress, which consists of several low-level cellular functions including response to heat, starvation, osmotic stress and topological incorrect protein. (**B**) New modularity paradigm. Colors represent modules.

Apparently, cellular processes are hierarchically organized, so does the biological networks consisting of interacting molecules that carry out cellular functions. The second question is about how higher-level cellular functions build by connecting low-level ones in biological networks. To answer this question, we studied a subnet related to cellular response to stress, which consists of several low-level cellular functions such as response to heat, starvation, osmotic stress and so on (Figure 5A). This subnet consists of both spoke-like topologies (i.e., nodes around gene *hog1, pho85* and *cdc48*) and a clique (i.e., members of nuclear pore complex such as *nup100, nup133, nup120* and *nup84*). From this subnet, one can find that functional modules need not be rigid, densely interconnected structures. Moreover, genes may belong to different modules at the same level of the hierarchy, which are in contrast to the “deterministic hierarchical model”. The overlap between functional modules is consist with the fact that genes are always multi-functional, which allows one function to influence another more effectively. For example, *cdc28* is not only a regulator in cellular response to stress (Figure 5A), but also a regulator in mitosis, which may conduct the interplay between environmental stress and cell cycle. It has been found that osmotic stress causes the down regulation of *cdc28* activity and causes a cell cycle delay in *Saccharomyces cerevisiae*[25]. To include these features, we introduce a continuous modularity paradigm (Figure 5B), where the border of a module and overlap between modules can both be found, allowing each module to accomplish a relatively autonomous function and to influence the function of other modules. This new paradigm is enriched of spoke-like topology; however, a few cliques can also be seen. A high-level functional module is built by connecting these overlapped modules together, and several high-level modules further build a higher-level module in a similar way. This procedure can be repeated to generate a hierarchical architecture. This new paradigm may not be as simple and concise as “deterministic hierarchical model”, but it takes into account the multi-functionality of biological molecules and flexibility of modular structure. Furthermore, it suggests that appropriate overlap could be a primary basis for a high-level cellular function to integrate information from its low-level modules and resolve conflicts between them.

## Conclusions

It is widely accepted that biological hierarchy can be well characterized by a “deterministic hierarchical model”, because it reconciles modularity and scale-freeness, with *C*(*k*) following a scaling law [7]. A later study further developed a more general power-law of *C*(*k*) to identify hierarchical network [23]. Although the model successfully shows that *C*(*k*) of a “deterministic hierarchical model” network follows the scaling law *C*(*k*) ~ *k*^-1^, there is no evidence showing that a network following this scaling law is necessarily a network of hierarchy. Therefore, it is not sufficient to identify network hierarchy. More evidences comes from the fact that many networks with no significant variation of *C*(*k*) are also hierarchically organized. It has been found that many complex systems have hierarchical organization, including social networks that are known to be assortative and lack the variation of *C*(*k*) [26,27]. These studies further suggest that the scaling of *C*(*k*) is neither a sufficient nor a needed condition for a network to be hierarchical. Although two former studies have suggested the shortcomings of using the variation of *C*(*k*) in assessing network hierarchy [10,26], our study provided further and more direct evidences. Nowadays, many sophisticated models have been developed to include the variation of *C*(*k*) and degree distribution. However, since the variation of *C*(*k*) is still widely used as a standard indicator of hierarchical network structure, it is necessary to specifically point out the limitations of “deterministic hierarchical model”. By doing this, our study suggests the need to reconsider the modularity nature of biological systems. In particular, we stress the importance of overlap in the communication of different modules. Our study may be applicable to other complex networks as well, such as WWW, of which the variation of *C*(*k*) was interpreted as the existence of network hierarchy too [23].

## Methods

### Datasets

Our analysis includes four types of biological networks of yeast: Physical protein interaction network, genetic synthetic lethal network, gene co-expression network and metabolic network. Dataset of protein-protein interaction was obtained from DIP (version 10/2010) [28]. To display the organization for the top 6 best connected nuclear proteins, a high-confidence dataset curated from literatures and high-throughput sources was used [24], where the subcellular localization information was according to MIPs annotation [29]. Dataset of synthetic lethal interaction was obtained from Biogrid (version 3.1.72) [30], and the metabolic networks of 43 organisms were obtained from Jeong H et al. [31]. The gene co-expression network was constructed according to the yeast cell cycle expression data [32]. Arrays where greater than 10% of the gene expression information was missing were removed and genes where more than 7 arrays the expression information was missing were removed. Then, the Pearson coefficient was calculated for every gene pair, and only gene pairs with absolute value larger than 0.65 were used to construct the gene co-expression network.

### Random networks

To generate seed networks that preserves the joint probability *P*(*k*_*i*_, *k*_*j*_), we draw N·P(k) nodes from the degree distribution *P*(*k*) for each degree k, and then form a node set S containing k_*i*_ copies of each node *i*, where *N* denotes the number of nodes in biological network. Then, we select at random two nodes from *S*, connect them to generate a new random network and then remove them from *S*. At each time, we estimate the joint probability *R*(*k*_*i*_, *k*_*j*_) in the random network, and test if $R(k_{i},k_{j})\leq P(k_{i},k_{j})$. When the condition is not fulfilled, we discard the two nodes and draw two new ones from *S*. This step is repeated until $R(k_{i},k_{j})==P(k_{i},k_{j})$ for all the degrees.

## Misc

Dapeng Hao and Cong Ren contributed equally to this work

## Competing interests

The author(s) declare that they have no competing interests.

## Authors’ contribution

DP and CL contributed to the design of the study and the writing of the manuscript. DP and CR performed the analysis in Figure 1, 2, 3,4 and Additional file 1: Figure S1, Additional file 2: Figure S2. Additional file 3: Figure S3, DP and CL contributed to the revision and Figure 5. CR contributed to the biological discussion in the manuscript. All authors read and approved the final manuscript.

## Supplementary Material

Additional file 1 **Figure S1.** Clustering coefficient distribution. (A) *C*(*k*) curves of protein interaction network and (B)genetic interaction network, as well as their random counterparts of same degree distribution (generated by randomly rewiring the edges, black circles). Click here for file

Additional file 2 **Figure S2.** The variation of *C*(*k*) of protein interaction network (red circles) and stringent but uncorrelated random network (black circles). The random network and the protein interaction network have at least 30% of edges overlapped. Click here for file

Additional file 3 **Figure S3.** The functional module associated to cell wall organization is built by connecting several spokes in yeast interactome. Click here for file
