# Supplementary figures and images for: Revisiting the variation of clustering coefficient of biological networks suggests new modular structure
Source: BMC Syst Biol. 2012 May 1;6:34. doi: 10.1186/1752-0509-6-34 (PMC3465239; doi:10.1186/1752-0509-6-34)

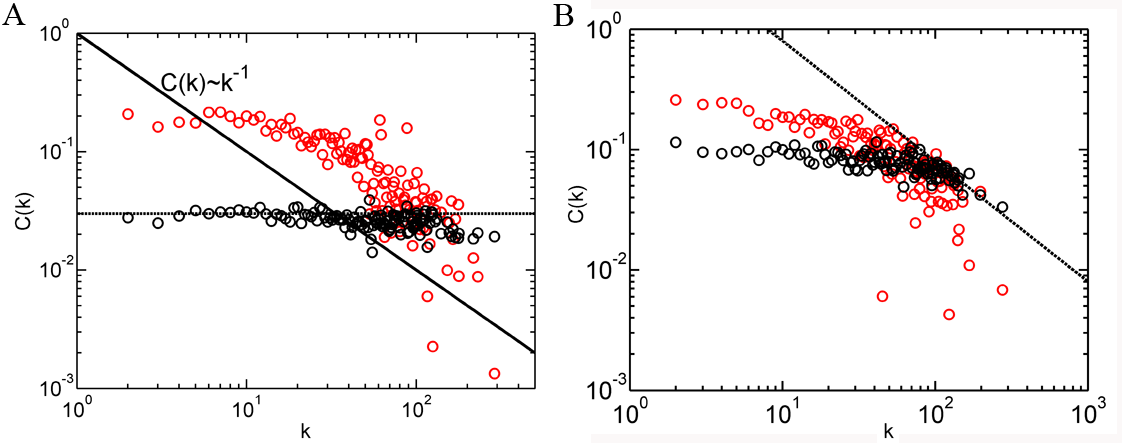

Supplement: Additional file 1 — Figure S1. Clustering coefficient distribution. (A) C(k) curves of protein interaction network and (B)genetic interaction network, as well as their random counterparts of same degree distribution (generated by randomly rewiring the edges, black circles). [file 1752-0509-6-34-S1.tiff]

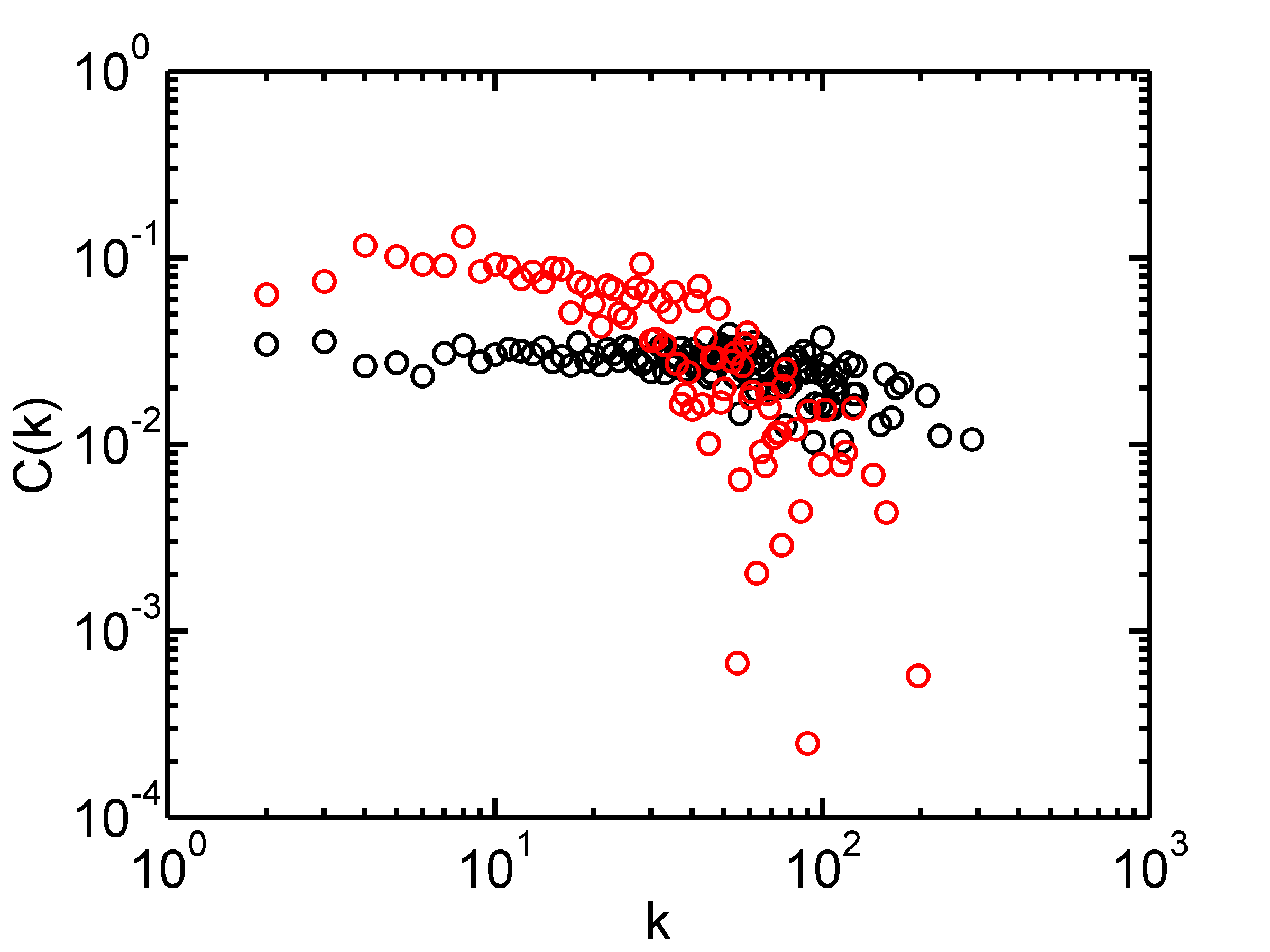

Supplement: Additional file 2 — Figure S2. The variation of C(k) of protein interaction network (red circles) and stringent but uncorrelated random network (black circles). The random network and the protein interaction network have at least 30% of edges overlapped. [file 1752-0509-6-34-S2.tiff]

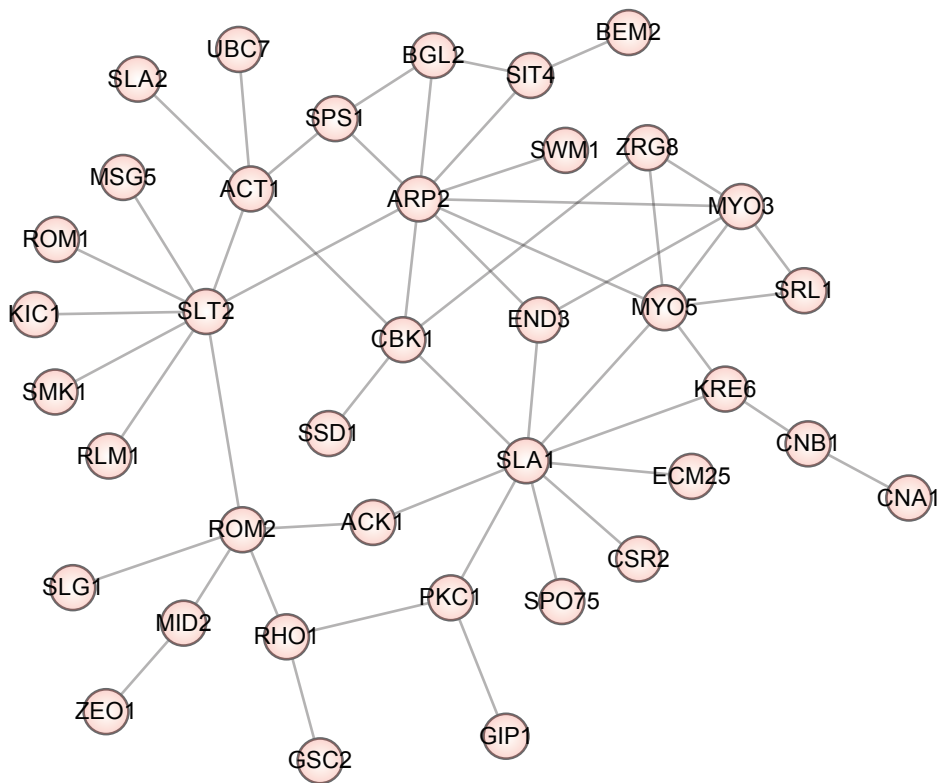

Cell wall organization

Supplement: Additional file 3 — Figure S3. The functional module associated to cell wall organization is built by connecting several spokes in yeast interactome. [file 1752-0509-6-34-S3.pdf]
